# Does a pay-for-performance program for primary care physicians alleviate health inequity in childhood vaccination rates?

**DOI:** 10.1186/s12939-015-0231-6

**Published:** 2015-12-14

**Authors:** Alan Katz, Jennifer Emily Enns, Dan Chateau, Lisa Lix, Doug Jutte, Jeanette Edwards, Marni Brownell, Colleen Metge, Nathan Nickel, Carole Taylor, Elaine Burland, The PATHS Equity Team

**Affiliations:** Manitoba Centre for Health Policy, 408-727 McDermot Ave, Winnipeg, MB R3E 3P5 Canada; Department of Community Health Sciences, University of Manitoba, S113-750 Bannatyne Ave, Winnipeg, MB R3E 0W3 Canada; School of Public Health, University of California, 50 University Hall, #7360, Berkeley, CA 94720-7360 USA; Winnipeg Regional Health Authority, Primary Health Care and Chronic Disease, 496 Hargrave St, Winnipeg, MB R3A 0X7 Canada; Winnipeg Regional Health Authority, 200-1155 Concordia Ave, Winnipeg, MB R2K 2M9 Canada

**Keywords:** Health equity, Income inequality, Childhood, Immunization, Vaccination, Manitoba, Canada

## Abstract

**Introduction:**

Childhood vaccination rates in Manitoba populations with low socioeconomic status (SES) fall significantly below the provincial average. This study examined the impact of a pay-for-performance (P4P) program called the Physician Integrated Network (PIN) on health inequity in childhood vaccination rates.

**Methods:**

The study used administrative data housed at the Manitoba Centre for Health Policy. We included all children born in Manitoba between 2003 and 2010 who were patients at PIN clinics receiving P4P funding matched with controls at non-participating clinics. We examined the rate of completion of the childhood primary vaccination series by age 2 across income quintiles (Q1–Q5). We estimated the distribution of income using the Gini coefficient, and calculated concentration indices for vaccination to determine whether the P4P program altered SES-related differences in vaccination completion. We compared these measures between study cohorts before and after implementation of the P4P program, and over the course of the P4P program in each cohort.

**Results:**

The PIN cohort included 6,185 children. Rates of vaccination completion at baseline were between 0.53 (Q1) and 0.69 (Q5). Inequality in income distribution was present at baseline and at study end in PIN and control cohorts. SES-related inequity in vaccination completion worsened in non-PIN clinics (difference in concentration index 0.037; 95 % CI 0.013, 0.060), but remained constant in P4P-funded clinics (difference in concentration index 0.006; 95 % CI 0.008, 0.021).

**Conclusions:**

The P4P program had a limited impact on vaccination rates and did not address health inequity.

## Introduction

Inequity in health outcomes is a global challenge that creates economic losses and health care burdens, such as loss of productivity and tax payments, higher welfare payments and health care costs [1–7]. Inequity in child health often translates into chronically poorer health in adulthood, adding further burdens on the health care system and the population [8]. Measures associated with socioeconomic status (SES), such as income, employment, and education, are only a few of the social determinants of health [2]. However, social programs addressing some of these determinants have been shown to be effective at alleviating health inequity in Canada [9] and globally [10]. Researchers at the Manitoba Centre for Health Policy (MCHP) are currently conducting multiple evaluations as part of the PATHS (Pathways To Health and Social Equity for Children) program of research, with the aim of determining the impact of established programs on health and social inequity in children in Manitoba [11]. Among these programs is the Physician Integrated Network (PIN), a primary care renewal initiative developed by the Ministry of Health in Manitoba, which aimed to improve primary care outcomes by providing clinics with pay-for-performance (P4P) funding [12].

The PIN program provided funding to clinics in Manitoba for meeting quality of care targets on selected clinical process indicators, including meeting childhood vaccination targets. The provincially recommended childhood vaccination program in Manitoba follows a vaccination schedule that stipulates a primary series be administered by age 2 [13]. The vaccinations have been shown to afford valuable protection against mortality from infectious disease in Canada [14], defending against all-cause mortality [15] and limiting exacerbation of chronic diseases such as asthma attacks [16, 17]. However, analyses of provincial vaccination data from 2002/03 and 2007/08 have demonstrated that childhood vaccination rates in low-SES Manitoba populations fall significantly below the provincial average [13]. Inequity in vaccination exists in other Canadian provinces and globally, and is associated with factors such as SES [18–24], household income [19, 21, 25, 26], mother’s knowledge about vaccinations [23, 27], length of maternity leave [23], and access to transportation [28].

Although the PIN program aimed to improve the overall quality of primary care and did not specifically target low-SES Manitobans, there is concern that P4P programs operating within the current pattern of health inequity can widen the socioeconomic gap. It has long been recognized that inequities left unaddressed have damaging effects on health outcomes in vulnerable populations [10]. Therefore, we evaluated the PIN P4P program to determine whether it alleviated SES-based inequity in childhood vaccination rates in Manitoba.

## Methods

### Administrative data sources

The PATHS Data Resource [11] is a de-identified collection of data which is part of the Population Health Research Data Repository (the Repository) housed at MCHP, University of Manitoba. The PATHS resource was created by linking information across several databases of population-based individual-level data using scrambled personal identifying numbers, such that it comprises health and social services use data for over 99 % of the children in Manitoba. In this study, we used three datasets within the PATHS resource: the Manitoba Immunization Monitoring System which contains vaccination dates and identifiers; claims for physician visits and tests ordered; and the Manitoba Health Registry which includes demographic data (e.g., sex, age, postal code) on virtually every child residing in Manitoba. The validity of the data included in the PATHS resource have been well documented [29–32]. The study was approved by the University of Manitoba Health Research Ethics Board (HREB) and the Manitoba Health Information Privacy Committee (HIPC).

Income quintiles were constructed using public-use census data for each dissemination area (~400-700 individuals) in Manitoba based on the postal code of the child at age 2. The dissemination areas were sorted by average income and divided into quintiles of equal population size. About 1 % of individuals in Manitoba were excluded from the income quintiles, because their postal code did not link with a dissemination area, their dissemination area had a suppressed average household income, or they lived in a dissemination area where 90 % or more of the population was institutionalized (i.e., personal care home, prison).

### Study cohorts

The PIN program was implemented in two phases: Phase 1 was launched in 2007 with the participation of four primary care clinics, and eight additional clinics were recruited for Phase 2 in 2008. The twelve clinics were distributed across Manitoba regional health authorities (RHAs) and included 170 physicians with over 180,000 patients assigned to their care during the period of study [33]. P4P funding was based on 15 care provision process indicators, including childhood immunizations, with quarterly extracts from the clinic electronic medical record providing evidence of clinical practice.

**Fig. 1:**
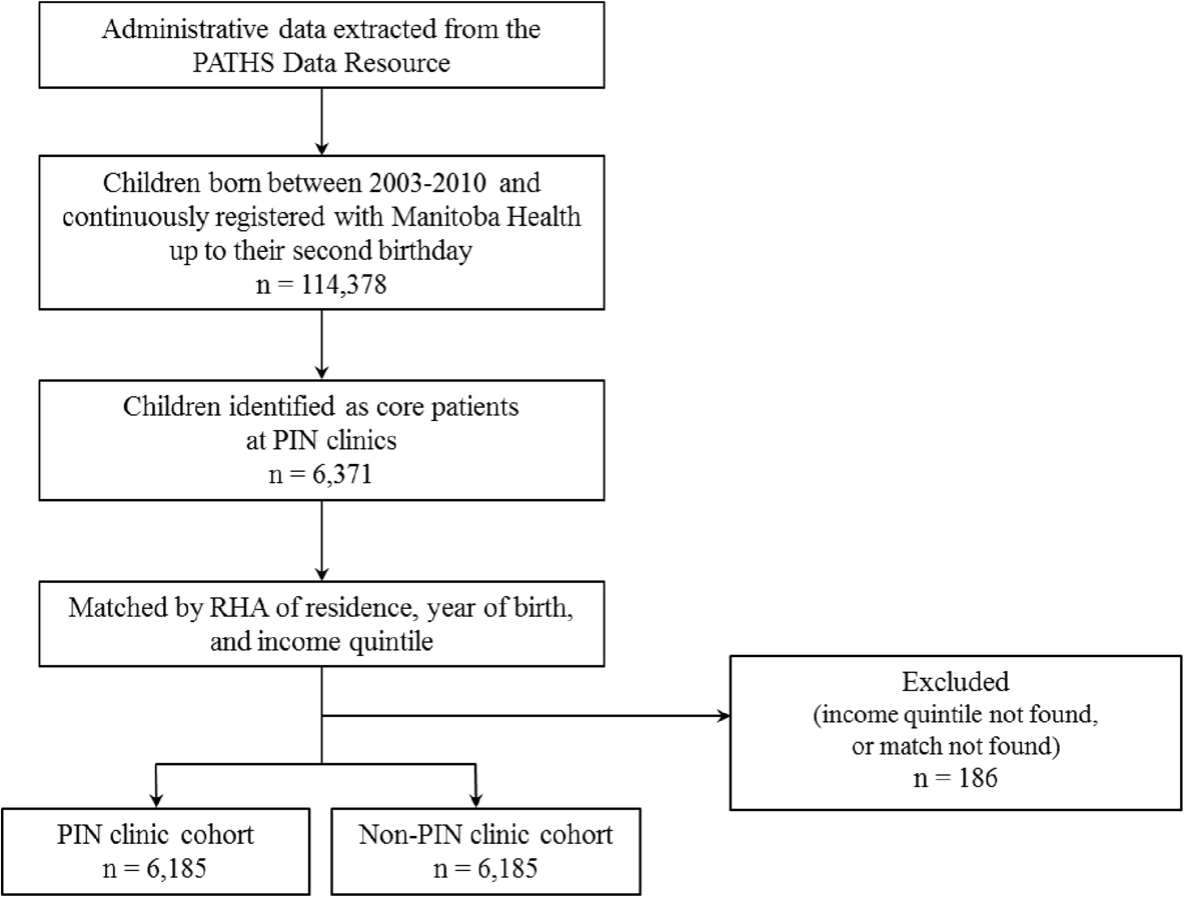
Flowchart depicting the creation of study cohorts from PATHS Data Resource administrative data

The development of the study cohorts is described in Fig. 1. PIN clinics first identified their core patients in their electronic medical records using an established algorithm [33]. Children were included in the PIN clinic cohort if they were born in Manitoba between 2003 and 2010, were continuously registered with Manitoba Health, Healthy Living and Seniors (MHHLS) up to their second birthday, and were identified as core PIN clinic patients. We matched these patients to non-PIN clinic controls by RHA of residence, income quintile and birth year.

### Primary outcomes

We determined the rate of vaccination completion by income quintile in each cohort by counting the number of vaccine doses children had received by the age of 2 and comparing these to the Manitoba Immunization Guidelines [12]. To measure the distribution of our health outcome (completion of vaccination) across income quintiles, we calculated the concentration index for the five-year study period, providing an estimate of SES-related inequity [34, 35]. The concentration index is estimated from the concentration curve, which is based on the proportion of people in a population at different SES levels with a particular outcome (in this case, vaccination). The concentration index is determined by calculating the area between the concentration curve and the line of equity (representing 100 % equal distribution of vaccination among SES quintiles), and thereby allows an estimation of SES-related inequity with values ranging from -1 to +1. A concentration index with a value of 0 would indicate that vaccination was distributed evenly across income quintiles. A concentration index approaching +1 would indicate that vaccination is concentrated among the wealthy quintiles, while a value approaching -1 would mean it is more common among the poor quintiles. Concentration indices were calculated for PIN and matched non-PIN cohorts before and after the PIN program was implemented to assess whether the PIN program was associated with reduced SES-related inequity in vaccinations.

Changes in concentration indices for vaccination could be influenced by corresponding changes in underlying income inequality [36]. We therefore estimated the Gini coefficient to quantify the distribution of family income over the study period [34, 35, 37]. The Gini is estimated from the Lorenz curve, a measure of wealth distribution within a population that demonstrates the percentage of people falling within a certain range of income. The Gini represents the area between the Lorenz curve and 100 % equality in income distribution, allowing the degree of income inequality to be quantified with values falling between 0 (equal distribution of income across the population) and 1 (absolute inequality, where all income belongs to a single individual). Gini coefficients were calculated before and after the PIN program was implemented in both PIN and non-PIN cohorts to determine whether income inequality changed over time in the study population.

We also estimated the Kakwani Progressivity Index (KPI). The KPI is defined as the difference between the concentration index (health inequity) and the Gini coefficient (income distribution), and ranges from -2 to 1 [38]. For our study, the KPI was modified (mKPI) so that mKPI = Gini - |concentration index|. Hence, a positive mKPI would occur when health inequity was less than income inequality, i.e., given the unequal distribution of income across the population (indicated by the Gini coefficient), health inequity is less than would be expected. Conversely, a negative mKPI would signify that health inequity was greater than expected, taking into account the underlying income inequality. Measures of precision (e.g., standard errors, 95 % confidence intervals [CI]) were estimated for the concentration indices, Gini coefficients and mKPIs using bootstrapping [39]. All data analyses for this paper were generated using SAS software, Version 9.3 [40].

## Results

Figure 1 shows the development of the study cohorts, and Table 1 lists the demographics for the children included in the study cohorts. The children were matched by birth year, RHA of residence, and income quintile; and although we didn’t match for sex, the proportions were very similar between groups.

**Table 1:** Demographics of the study population

	PIN clinic cohort^a^
	No. (%)
*N*	6, 185
Birth Year	
2003	117 (1.9)
2004	610 (9.9)
2005	956 (15.5)
2006	1, 494 (24.2)
2007	1, 548 (25.0)
2008	913 (14.8)
2009	547 (8.8)
Sex	
Male	3, 182 (51.4)
Female	3, 003 (48.6)
RHA	
Interlake-Eastern	115 (1.9)
Northern	20 (0.3)
Southern	4, 026 (65.1)
Prairie Mountain (Western)	1, 036 (16.8)
Winnipeg/Brandon	988 (16.0)
Income Quintile	
Q1 (lowest)	865 (14.0)
Q2	1, 132 (18.3)
Q3	1, 738 (28.1)
Q4	1, 538 (24.9)
Q5 (highest)	912 (14.7)

*RHA* regional health authority

^a^The PIN and non-PIN clinic cohorts were matched on birth year, RHA and income quintile. In the non-PIN clinic cohort, there were 3,161 (51.1 %) males and 3,024 (48.9 %) females

**Fig. 2:**
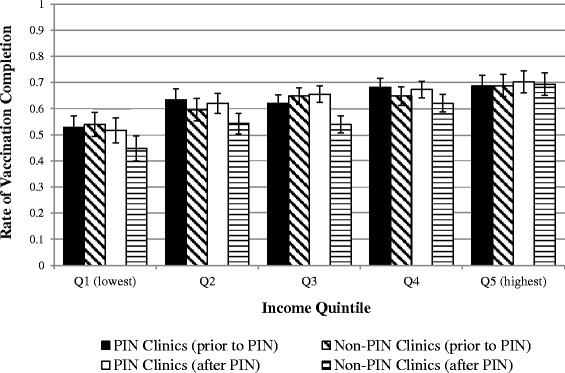
Rates of vaccination completion at age 2 by income quintile. Error bars indicate 95 % CIs

Figure 2 shows the rates of vaccination completion by income quintile for the study cohorts before and after the PIN program. Children in the higher income quintiles were more likely to have completed the primary vaccination series than those in the lower income quintiles. In the non-PIN clinic cohort, vaccination completion rates declined in children in Q2 and Q3 over the course of the PIN program.

Table 2 presents measures of the distribution of income and SES-related differences in vaccination for the study cohorts before and after implementation of the PIN program. At baseline, inequality in income distribution existed in both groups, as the Gini coefficients were significantly higher than 0 in the PIN clinics and non-PIN clinics. Concentration indices for the PIN clinics and the non-PIN clinics were significantly higher than 0, indicating that there was health inequity in vaccination present in both groups, that is, individuals with higher income were more likely to be vaccinated than those with lower income. The mKPI in both groups was also significantly greater than 0 at baseline, indicating that although health inequity was present, it was less than expected given the underlying income inequality.

**Table 2:** Measures of income distribution and SES-related inequity in vaccination completion

		PIN clinics	Non-PIN clinics	Difference between groups
		(95 % CI)	(95 % CI)	(95 % CI)
Prior to PIN	Gini	0.135 (0.127, 0.142)	0.154 (0.147, 0.160)	0.019 (0.010, 0.028)
	Conc index	0.036 (0.019, 0.053)	0.031 (0.016, 0.045)	0.005 (-0.011, 0.022)
	KPI	0.099 (0.080, 0.117)	0.123 (0.108, 0.138)	−0.024 (−0.048,−0.001)
After PIN	Gini	0.136 (0.129, 0.143)	0.152 (0.146, 0.158)	0.016 (0.006, 0.025)
	Conc index	0.042 (0.027, 0.057)	0.067 (0.048, 0.086)	0.025 (0.002, 0.049)
	KPI	0.094 (0.077, 0.111)	0.084 (0.066, 0.103)	0.010 (−0.008, 0.027)

*CI* confidence interval, *Conc Index* concentration index, *KPI* Kakwani Progressivity Index

At the end of the 5-year PIN program, Gini coefficients in the PIN clinics and non-PIN clinics remained significantly higher than 0, signifying that a significant difference in income distribution between the two groups persisted. Concentration indices for the PIN clinics and the non-PIN clinics remained significantly higher than 0, indicating that health inequity in vaccination continued in both groups after the PIN intervention. The mKPI showed that health inequity was less than expected when accounting for the underlying income inequality in these groups.

In Table 3, we show the changes in health equity measures between the start and the end of the 5-year PIN program (i.e., difference over time). The Gini did not change significantly in either cohort over the course of the PIN program. The concentration index for the non-PIN clinic cohort increased significantly, indicating that health inequity in vaccination completion worsened over time in this cohort. In other words, in clinics that did not have P4P funding, the gap in vaccination rates between wealthy and low-income families widened over the study period. In this same cohort, mKPI decreased significantly over time, indicating that by the end of the study period, any factors mitigating the impact of the income inequality on health inequality had weakened.

**Table 3:** Changes in income inequality and health inequity measures over time

	PIN clinics	Non-PIN clinics
	Difference over time (95 % CI)	Difference over time (95 % CI)
Gini coefficient	0.002 (−0.004, 0.007)	−0.002 (−0.004, 0.008)
Concentration index	0.006 (−0.008, 0.021)	0.037 (0.013, 0.060)
mKPI	−0.005 (−0.010, 0.019)	−0.039 (-0.064, -0.014)

*CI* confidence interval, *mKPI* modified Kakwani Progressivity Index

## Discussion

The findings of this study suggest that the PIN program helped to maintain the pre-intervention level of inequity in childhood vaccination rates by the age of 2. Although income distribution remained unequal in the both the PIN and non-PIN groups throughout the course of the P4P intervention, inequities in vaccination across income quintiles worsened in non-PIN clinics while remaining stable in PIN clinics. We expected to see a decrease in health inequity in the PIN clinics, but this was offset by the health inequity increase in the non-PIN clinics.

In the last decade, P4P programs have become a popular method of encouraging improved primary health care, although it is still uncertain how they impact patient care and disease management [41, 42]. With regard to vaccinations specifically, these programs have produced mixed results. A study in Ontario demonstrated that a P4P program had no effect on childhood immunizations, and only modest effects on other indicators such as mammograms and colorectal cancer screening [43]. An analysis in the United States measured the effect of P4P programs on health care quality, including childhood vaccination [44]; however, the study failed to find evidence that P4P initiatives brought about major improvements in quality of care. Another study found that P4P funding modestly improved childhood vaccination rates [45].

To our knowledge, our study is the first to examine whether a P4P intervention can effectively alleviate SES-related health inequities in vaccination. Our findings suggest that in the absence of a P4P funding initiative, inequity in vaccination completion increased in the general population. Although the PIN program mitigated the increasing negative effect of low SES on vaccination during the study period, the program did not address the pre-existing health inequity. The social determinants of health, including SES, have a direct effect on the health of individuals and populations, and disparities in health affect the health status of the overall population [46, 47]. Although P4P funding models may provide incentive to clinicians to improve the quality of care they offer to patients, other research has shown that they do little to address health equity gaps [48–50], unless the equity gap is extreme. Our results demonstrated a positive effect compared to the control group, but did not show an absolute reduction in inequity. The relatively small gap in vaccination completion rates in Manitoba (Fig. 2) may have contributed to the PIN program’s lack of effect on health equity, since there is more potential for improving low-SES patient outcomes where a large equity gap exists.

### Strengths and limitations

Strengths of our study include the ability to link administrative data across several databases, allowing us to capture virtually all of the eligible population for our study cohort, including rural and urban populations. Although our findings are specific to the primary care renewal initiatives implemented in the province of Manitoba, the inclusion of different geographic regions lends generalizability to the results for the entire population of the province. The analysis was limited by the use of area-level income data. However, studies have shown that area-level measurements, such as those collected from dissemination areas, provide a good approximation of individual-level SES [51]. Although we detected a significant gap in health equity over time, the absolute change in the concentration index between groups was very small; this is common for outcomes (such as vaccination completion) where the health equity gradient from low- to high-income is narrow at baseline.

## Conclusions

Overall, our study suggests that a P4P incentive program was effective at maintaining SES-related health equity in vaccination completion, but did not improve it. Given how few studies address equity in the delivery of primary care, future research should take into consideration not only whether primary care renewal initiatives have an impact on health, but also the role of social determinants in driving inequities in health and health care use.

## Abbreviations

CI: Confidence interval
HIPC: Health Information Privacy Committee
HREB: Health Research Ethics Board
KPI: Kakwani Progressivity Index
MCHP: Manitoba Centre for Health Policy
MHHLS: Manitoba Health, Healthy Living and Seniors
mKPI: modified KPI
PIN: Physician Integrated Network
PATHS: Pathways to Health and Social Equity for Children
P4P: Pay-for-performance
SES: Socioeconomic status
